# Ultra-short bowel syndrome after near-total small bowel resection for gangrenous gut due to superior mesenteric artery thrombosis: a case report

**DOI:** 10.1093/jscr/rjag738

**Published:** 2026-10-08

**Authors:** Faran Hamid, Bilal Afsar, Afsar Ali Bhatti, Amir Usman, Naila Yasmeen, Dania Hussain, Anas Nasir, Bilal Aslam, Fazeela Bibi, Mahshid Muhammadi

**Affiliations:** Department of Surgery, University of Lahore, 1-Km Defence Road, Bhuptian Chowk, Lahore 54792, Pakistan; Department of Surgery, Sheikh Zayed Hospital, Block D, New Muslim Town, Lahore 54600, Punjab, Pakistan; Department of Surgery, Doctors Hospital Lahore, 152-G/1, Canal Bank, Block G1, Phase 1, Johar Town, Lahore 54590, Pakistan; Department of Surgery, University of Lahore Teaching Hospital Lahore, Defence Road, Bhuptian Chowk, Lahore 54792, Pakistan; Department of Surgery, Doctors Hospital Lahore, 152-G/1, Canal Bank, Block G1, Phase 1, Johar Town, Lahore 54590, Pakistan; Department of Surgery, United Medical and Dental College, UMDC Road, Sector 48-H, Korangi Creek, Karachi 75190, Pakistan; Department of Surgery, Sheikh Zayed Medical College, Block D, New Muslim Town, Lahore 54600, Punjab, Pakistan; Department of Surgery, University of Lahore, 1-Km Defence Road, Bhuptian Chowk, Lahore 54792, Pakistan; Department of Surgery, Jinnah Medical and Dental College, 22-23 Shaheed-e-Millat Road, BMCHS Sharafabad, Karachi 74000, Pakistan; Department of Surgery, Kabul University of Medical Sciences Abu Ali Ibn Sina, 3rd District, Kabul 1001, Afghanistan

**Keywords:** ultra-short bowel syndrome, superior mesenteric artery thrombosis, mesenteric ischaemia, small bowel resection, total parenteral nutrition, intestinal adaptation

## Abstract

Ultra-short bowel syndrome (USBS) is a rare and life-threatening condition defined by the retention of ˂30 cm of small intestine following resection. We report the case of a 53-year-old male who presented with gangrenous gut secondary to superior mesenteric artery thrombosis, necessitating near-total small bowel resection. The patient was left with ~15 cm of proximal jejunum and 10 cm of distal ileum, with a jejuno-ileal end-to-end anastomosis. He developed severe postoperative watery diarrhoea and required Total Parenteral Nutrition (TPN) via a peripherally inserted central catheter line. With careful multidisciplinary management, the patient achieved intestinal adaptation and was weaned off TPN ~6 weeks postoperatively. This case highlights the challenges of managing USBS in a resource-limited setting and demonstrates that survival with gut adaptation is possible even with very limited residual small bowel length, provided that experienced surgical care and nutritional support are available.

## Introduction

The length of the small intestine ranges between 3 m and 10 m with an average of 6.5 m [1]. It is made up of the duodenum (25 cm), jejunum (1.5 m), and ileum (distal three-fifths). Resection of up to a third or even half is compatible with a normal life. However, survival has been reported with a small bowel length of only 45 cm [2]. A major resection leads to short gut syndrome with malabsorption of macro- and micro-nutrients [3]. Long-term survival is enhanced with the use of parenteral nutrition [4], but there are limited publications reporting survival without parenteral nutrition.

## Case presentation

The case is of a 53-year-old male resident of Sargodha (Punjab), Pakistan. He remained admitted in a private hospital at Sargodha City with complaints of abdominal pain, abdominal distension, vomiting, and diarrhoea. A computed tomography (CT) scan was carried out at Sargodha City which revealed ‘Superior Mesenteric Artery Thrombosis with small gut dilatation up to the level of the distal segment of ileum’.

The patient was referred to Doctors Hospital & Medical Centre Lahore for tertiary care management on 12 January 2025. He was a 53-year-old man who looked unwell, lying motionless, in severe pain, very pale, not jaundiced, with no lymphadenopathy but mildly dehydrated. His blood pressure was 106/70 mmHg, pulse rate was 133/min, respiratory rate was 21/min and temperature was 37.7°C. The abdomen was symmetrical, not moving with respiration, with generalized tenderness, guarding, rebound tenderness, board-like rigidity, and absent bowel sounds. The rectum was empty on digital examination. The patient was a known case of hypertension for the preceding 12 years and diabetes mellitus for the preceding 6 years.

Given the CT scan findings of superior mesenteric artery (SMA) thrombosis, the patient was diagnosed with mesenteric and gut ischaemia requiring urgent surgical intervention.

### Intraoperative management

After ~4 h of resuscitative measures, an emergency laparoscopy and proceed procedure was carried out via an infra-umbilical port under general anaesthesia. All the gut was analyzed and the major portion was found to be ischaemic; it was therefore decided to convert to an open midline laparotomy. Almost all of the small bowel, sparing the initial 15 cm of jejunum, was ischaemic up to the distal ileum, sparing only 10 cm of ileum from the ileocaecal junction.

During near-total small bowel resection, the mesentery, and thrombosed vessels were ligated, followed by a tension-free hand-sewn jejunoileal anastomosis, leaving 15 cm of jejunum and 10 cm of distal ileum. Intraoperative heparinization was started, the abdomen was irrigated with warm saline, and closure was completed with a pelvic drain placement. The intraoperative findings are illustrated in (Fig. 1).

**Figure 1:**
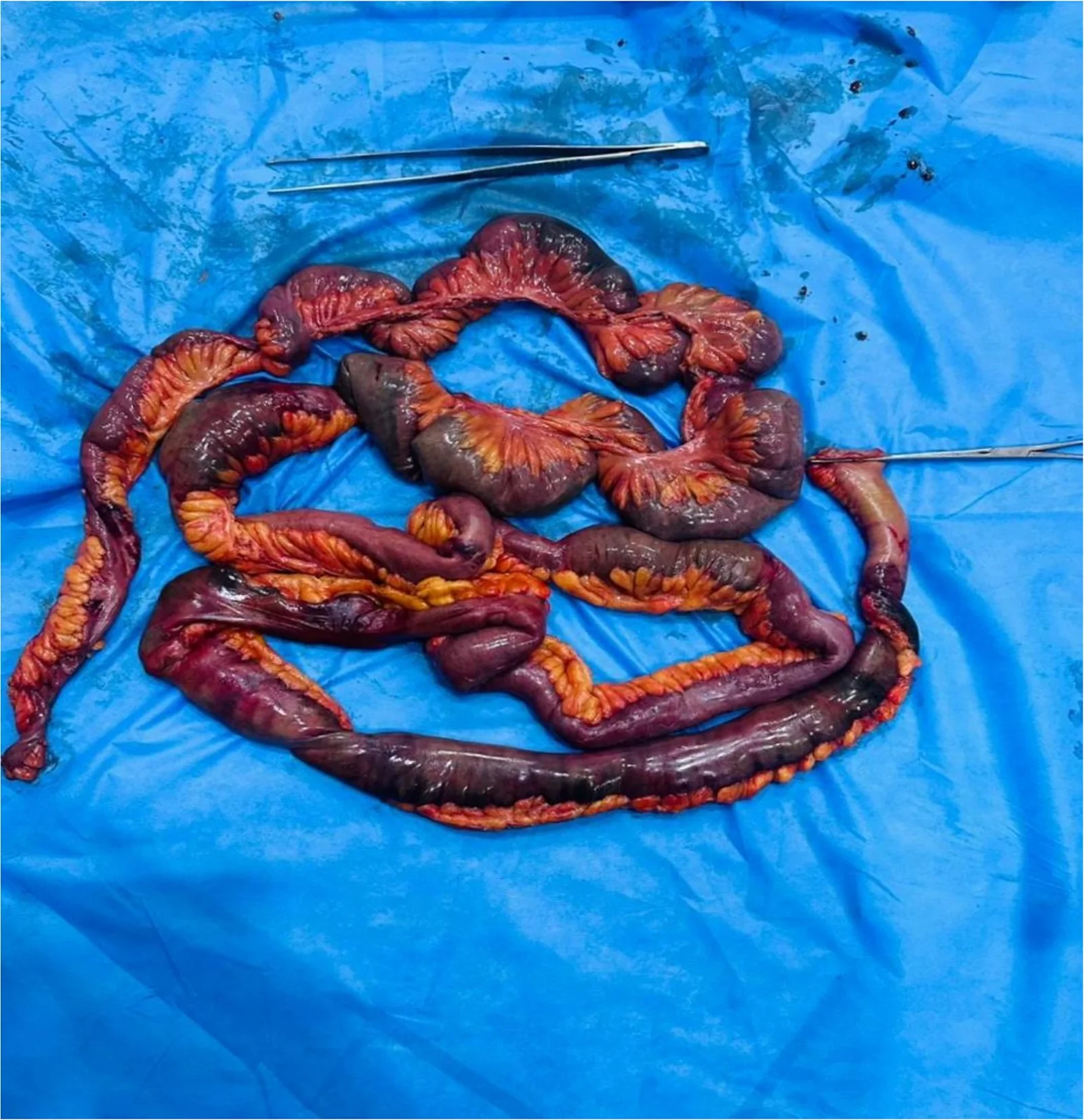
Intraoperative photograph demonstrating gangrenous bowel involving the majority of the small intestine due to SMA thrombosis.

### Postoperative management and progress

Proton pump inhibitor (intravenous pantoprazole 40 mg daily for 1 week) to lessen gastric hypersecretion, intravenous fluids (Ringer’s lactate 1.5 L in 24 h, 10% dextrose saline 1.5 L in 24 h), antibiotics (intravenous meropenem 1 g three times daily for 1 week, metronidazole 50 mg three times daily for 1 week), and analgesia (intravenous tramadol 30 mg three times daily for 1 week). TPN, or total parenteral nutrition, was started. However, he experienced up to 20 episodes of watery diarrhoea per day on days 4–6, which were managed with antidiarrheal medications. The skin sutures were taken out after the patient experienced a superficial surgical site infection and some wound dehiscence on the seventh postoperative day. With conservative treatment, the diarrhoea had subsided by the ninth postoperative day. After being kept on a peripherally inserted central catheter line in the right arm, the patient was released on TPN, oral anticoagulation (rivaroxaban 15 mg twice daily), other supportive care, and daily wound dressing recommendations.

Approximately 6 weeks after surgery, he was tolerating an oral diet only and TPN was discontinued. Oral vitamin and mineral supplementation were continued [5].

## Discussion

The near-total resection of the small intestine in this patient resulted in ultra-short bowel syndrome (USBS), with approximately ˂30 cm of small bowel remaining [6]. Short bowel syndrome is associated with reduced quality of life and a mortality of 10%–15% over 5 years [7]. Several challenges were encountered in the management of this patient.

### Diagnostic

The investigative and diagnostic capabilities of the hospital laboratory and radiology services were limited due to equipment malfunction and financial constraints.

### Economic

TPN is not generally available in many centres in Pakistan. Even when obtainable, it is very expensive (approximately USD 300 per day) and beyond the reach of most patients.

## Conclusion

This case report demonstrates that survival from ultra-short bowel syndrome is possible even in resource-limited settings, provided that expert surgical management and adequate nutritional support are available. TPN played a pivotal role in bridging the patient through the period of gut adaptation. Clinicians in low- and middle-income countries should be aware of these challenges when managing such complex cases and should advocate for improved access to parenteral nutrition and intestinal rehabilitation programmes.

## Data Availability

The data was taken from a patient who presented to our hospital, all data and references are publicly available on databases such as Pub-med and Google Scholar.

## References

[1] Ellis H, Mahadevan V. Clinical Anatomy: Applied Anatomy for Students and Junior Doctors. Chichester: Wiley-Blackwell, 2018.

[2] Liu MY, Tang HC, Yang HL et al. A short bowel (small intestine = 40 cm), no ileocecal valve, and colonic inertia patient works well with oral intake alone without parenteral nutrition. Case Rep Surg 2014:Article ID 290104;2014:1–3. 10.1155/2014/387307 PMC4082853 25018889

[3] O’Keefe SJ, Buchman AL, Fishbein TM et al. Short bowel syndrome and intestinal failure: consensus definitions and overview. Clin Gastroenterol Hepatol 2006;4:6–10. 10.1016/j.cgh.2005.10.002 16431298

[4] Wilmore DW, Dudrick SJ. Growth and development of an infant receiving all nutrients exclusively by vein. JAMA 1968;203:860–4. 10.1001/jama.1968.03140100042009 4965871

[5] Sala D, Chomto S, Hill S. Long-term outcomes of short bowel syndrome requiring long-term/home intravenous nutrition compared in children with gastroschisis and those with volvulus. Transplant Proc 2010;42:5–8. 10.1016/j.transproceed.2009.12.033 20172269

[6] Dore M, Junco PT, Moreno AA et al. Ultrashort bowel syndrome outcome in children treated in a multidisciplinary intestinal rehabilitation unit. Eur J Pediatr Surg 2017;27:116–20. 10.1055/s-0036-1597812 28052307

[7] DeLegge M, Alsolaiman MM, Barbour E et al. Short bowel syndrome: parenteral nutrition versus intestinal transplantation. Where are we today? Dig Dis Sci 2007;52:876–92. 10.1007/s10620-006-9416-6 17380398

